# Development of a PET Insert for simultaneously small animal PET/MRI

**DOI:** 10.1186/2197-7364-2-S1-A21

**Published:** 2015-05-18

**Authors:** Yingjie Wang, Zhiming Zhang, Daowu Li, Shuangquan Liu, Peilin Wang, Baotong Feng, Pei Chai, Long Wei

**Affiliations:** Division of Nuclear Technology and Applications, Institute of High Energy Physics, Chinese Academy of Sciences, Beijing, 100049 China; Beijing Engineering Research Center of Radiographic Techniques and Equipment, Beijing, 100049 China

PET/MR is a new multi-modality imaging system which provide both structural and functional information with good soft tissue imaging ability and no ionizing radiation. In recent years, PET/MR is under major progress because of the development of silicon photomultipliers (SiPM). The goal of this study is to develop a MRI compatible PET insert based on SiPM and LYSO scintillator. The PET system was constituted by the detector ring, electronics and software. The detector ring consists of 16 detector module. The inner diameter of the ring was 151 mm, the external diameter was 216 mm, which was big enough for small animal research, e.g. rat, rabbit and tupaia. The sensor of each module was 2*2 SensL SPMArraySL, coupled with an array of 14 x 14 LYSO crystals, each crystal measuring 2 mm x 2 mm 10 mm. The detector was encapsulated in a copper box for light and magnetic shielding. Resister charge multiplexing circuit was used in the front end electronics. Each detector output 8X and 8Y position signals. One summed timing signal was extracted from the common cathode of all 64 channels. All these signals were transmitted to digital electronic board by a 3 m long coaxial cable from inside of the MR to the outside. Each digital electronic board handled 8 detector modules based on FPGA to obtain the timing, position and energy information of a single event. And then these single events were sent to the coincidence processing board to produce coincidence packets which are prepared for further processing. A 0.2mCi 68Ge line source was used to do the preliminary imaging test. The image was reconstructed by 3D-OSEM algorithm. The initial result proved the system to be feasible as a PET. FDG phantom imaging and simultaneous PET/MR imaging are in progress.

